# HIF1*α*-Induced Glycolysis Metabolism Is Essential to the Activation of Inflammatory Macrophages

**DOI:** 10.1155/2017/9029327

**Published:** 2017-12-13

**Authors:** Ting Wang, Huiying Liu, Guan Lian, Song-Yang Zhang, Xian Wang, Changtao Jiang

**Affiliations:** Department of Physiology and Pathophysiology, Key Laboratory of Molecular Cardiovascular Sciences, Ministry of Education, School of Basic Medical Sciences, Peking University, Beijing 100191, China

## Abstract

Hypoxia-inducible factor (HIF) 1*α* is a metabolic regulator that plays an important role in immunologic responses. Previous studies have demonstrated that HIF1*α* participates in the M1 polarization of macrophages. To clarify the mechanism of HIF1*α*-induced polarization of M1 macrophage, myeloid-specific HIF1*α* overexpression (Lysm HIF1*α* lsl) mice were employed and the bone marrow-derived and peritoneal macrophages were isolated. RT-PCR results revealed that HIF1*α* overexpression macrophage had a hyperinflammatory state characterized by the upregulation of M1 markers. Cellular bioenergetics analysis showed lower cellular oxygen consumption rates in the Lysm HIF1*α* lsl mice. Metabolomics studies showed that HIF1*α* overexpression led to increased glycolysis and pentose phosphate pathway intermediates. Further results revealed that macrophage M1 polarization, induced by HIF1*α* overexpression, was via upregulating the mRNA expression of the genes related to the glycolysis metabolism. Our results indicate that HIF1*α* promoted macrophage glycolysis metabolism, which induced M1 polarization in mice.

## 1. Introduction

Macrophages are the main component of innate immunity and play important roles in various inflammatory diseases, including hepatitis, vascular diseases, inflammatory bowel diseases, rheumatoid arthritis, and airway inflammation [1–5]. Activated macrophages are commonly divided into two polarized phenotypes, classically activated M1 and alternatively activated M2. Macrophages activated by interferon *γ* or toll-like receptor agonists polarize to the M1 phenotype [6], which are proinflammatory macrophages and play a central role in the host's defense against infection and inflammatory diseases [7, 8]. Macrophages activated by Th2 cytokines, IL-4, and IL-13 are polarized to M2 phenotype, which are associated with inflammation relief and tissue remodeling [9, 10]. Macrophage activation can be altered by disrupting cellular energy metabolism [11, 12]. Recent studies have demonstrated that M1 macrophages demand glycolysis, while M2 macrophages require fatty acid oxidation [13, 14]. However, the metabolomics profiling and the metabolic mechanism of macrophages polarization remained undefined.

Hypoxia-inducible factor 1 (HIF1) has emerged as one of the central regulators of inflammation mediated by myeloid cells [15, 16]. HIF1 is an *α* and *β* heterodimer [15, 17]. Whereas HIF1*β* is constitutively expressed in cells regardless of O_2_ tension [18], HIF1*α* protein increases exponentially in response to reduced O_2_ concentration [19]. HIF1 has displayed a significant role in regulating cellular ATP concentration and myeloid cell function including cell aggregation, motility, invasiveness, and bacterial killing [20–22]. Importantly, it has been reported that HIF1 participates in the regulation of macrophage polarization [20]. As glucose metabolism determines polarization of macrophages [23, 24], whether glucose metabolism is involved in HIF1*α*-induced macrophage polarization process has remained unclear.

## 2. Materials and Methods

### 2.1. Chemicals and Reagents

RPMI 1640 medium was purchased from Gibco. Fetal Bovine Serum (FBS), penicillin, and streptomycin were purchased from HyClone. GM-CSF was purchased from PeproTech. Ammonium acetate, LPS, oligomycin, carbonyl cyanide p-trifluoromethoxyphenylhydrazone (FCCP), rotenone, and antimycin A were purchased from Sigma. BBL™ Thioglycollate Medium was purchased from BD Biosciences, US. [5-13C]glutamine was obtained from Cambridge Isotope Laboratories. HPLC grade ammonium hydroxide, acetonitrile, and methanol were purchased from Fisher Scientific. Deionized water was produced by a Milli-Q system.

### 2.2. Animals

Lsl-HIF1 dPA mice were obtained as described previously [25]. For myeloid-specific HIF1*α* overexpression, Lsl-HIF1 dPA mice were crossed with mice harboring the Cre recombinase under control of the lysozyme M (Lysm) promoter, which is found only in myeloid lineage cells, to obtain the Lysm HIF1*α* lsl mice. The wild-type (WT) and Lysm HIF1*α* lsl mice were littermate and on a C57BL/6 J background, after backcrossing with C57BL/6J mice for over ten generations. All the animal protocols were approved by the Animal Care and Use Committee of Peking University.

### 2.3. Peritoneal Macrophage

WT and Lysm HIF1*α* lsl mice (6- to 8-weeks old) were injected intraperitoneally with 4% thioglycollate solution (2 ml). Three days later, peritoneal cells were harvested by injecting the peritoneal cavity with PBS containing 10% FBS. Primary peritoneal macrophages were cultured with RPMI-1640 medium supplemented with 10% FBS. Medium was changed 2–4 h later. Thioglycollate-elicited peritoneal macrophages were attached on plates and continued culturing for 6 to 24 h.

### 2.4. Bone Marrow-Derived Macrophages (BMDMs)

Bone marrow cells were collected from WT and Lysm HIF1*α* lsl mice (4- to 6-weeks old). Adherent macrophages were cultured for 3 days in RPMI-1640 supplemented with 10% FBS and GM-CSF (10 ng/mL). Then, the medium was changed and the attached macrophages were obtained after another 3 days. To obtain the M1 polarization, macrophages were continued culturing for 2 days in RPMI-1640 supplemented with 10% FBS and LPS (10 ng/mL).

### 2.5. Quantitative RT-PCR

Total RNA was isolated from peritoneal macrophages or BMDMs using TRIzol reagent. cDNA was obtained using the M-MLV reverse transcriptase kit according to the manufacturer's instructions. RT-PCR amplification was performed using an Mx3000 Multiplex Quantitative PCR System and SYBR Green I reagent. Gene expression levels were normalized to the internal control 18S rRNA.

### 2.6. Extracellular Flux Analysis

An XF24 Extracellular Flux Analyzer was used to measure the respiratory conditions of murine peritoneal macrophages. Cells were plated at 5 × 10^4^ cells/well in 24-well XF microplates and cultured for 6 h. RPMI-1640 medium was replaced with XF base medium supplemented with 25 mM glucose and 2 mM pyruvate. After 1 h of incubation in a CO_2_-free incubator at 37°C, the oxygen consumption rate (OCR) and extracellular acidification rates (ECAR) were measured following the manufacturer's instruction. Mitochondrial stress tests were performed under basal conditions or with the treatment of metabolic reagents, including 1 mM oligomycin, 1 mM FCCP, 1 mM rotenone, and 1 mM antimycin A. ECAR was calculated by Wave software.

### 2.7. Metabolomics Analysis

Analysis of metabolites was performed with a liquid chromatography-tandem mass spectrometry. For metabolite extraction, cultured cells were washed with saline twice, lysed in 80% aqueous methanol (*v*/*v*), and equilibrated at −80°C for 20 min. [5-13C]glutamine was added as an internal standard. Cells were oscillated for 10 min and centrifuged with the speed of 14,000*g* for 10 min at 4°C. Cell supernatants of metabolite extracts were collected, dried, and stored at −80°C before injection.

For liquid chromatography-tandem mass spectrometry (LC-MS/MS) analysis, samples were reconstituted in water and analyzed using a QTRAP 5500 LC-MS/MS system (AB SCIEX) coupled with an ACQUITY UHPLC System (Waters Corporation). An Xbridge Amide column (100 × 4.6 mm i.d., 3.5 Lm; Waters Corporation) was employed for compound separation at 30°C. The mobile phase A was 5 mM ammonium acetate in water with 5% acetonitrile, and mobile phase B was acetonitrile. The linear gradient used was as follows: 0 min, 90% B; 1.5 min, 85% B; 5.5 min, 35% B; 10 min, 35% B; 10.5 min, 35% B; 14.5 min, 35% B; 15 min, 85% B; and 20 min, 85% B. The flow rate was 0.5 ml/min. MultiQuant v3.0 software (AB SCIEX) was used to process all raw liquid chromatography-mass spectrometry data and integrate chromatographic peaks. Integrated peak areas corresponding to metabolite concentrations were further analyzed using the MetaboAnalyst website (http://www.metaboanalyst.ca). Metabolite abundance was expressed relative to the internal standard.

### 2.8. Statistical Analysis

All data are presented as the mean ± SEM. Comparisons of data sets were performed using unpaired Student's *t*-tests for comparing two groups. Statistical analyses were performed using GraphPad Prism (GraphPad Software). A *P* value at ^∗^*P* < 0.05 and ^∗∗^*P* < 0.01 was considered statistically significant for all experiments.

## 3. Results

### 3.1. HIF1*α* Induced M1 Polarization of Macrophages

**Figure 1:**
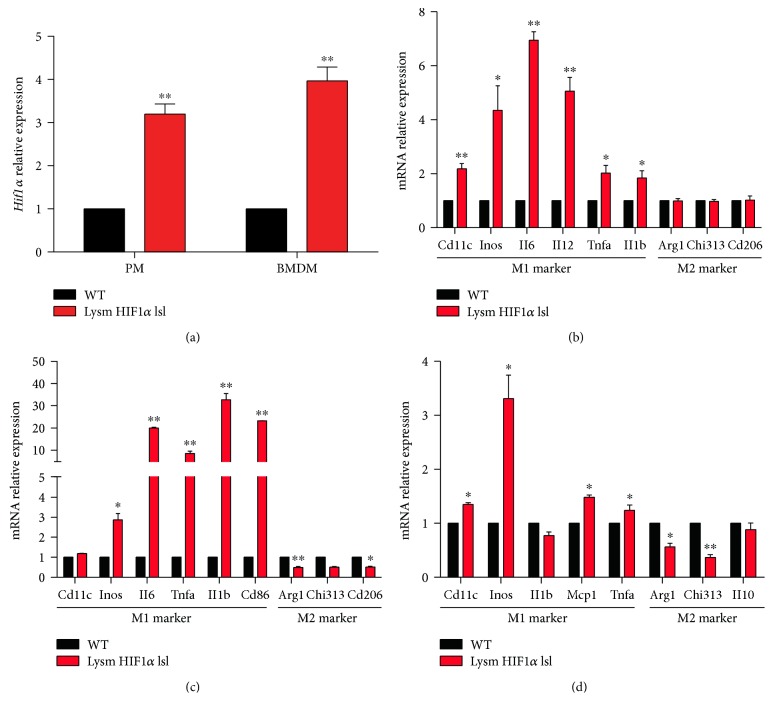
HIF1*α* induced M1 polarization of macrophages. (a) The relative mRNA level of HIF1*α* in the BMDMs and peritoneal macrophages of WT mice and Lysm Hif1*α* lsl mice. (b) The relative mRNA levels of M1 and M2 markers in the peritoneal macrophages isolated from WT mice and Lysm HIF1*α* lsl mice for 6 h. (c) The relative mRNA levels of M1 and M2 markers in the peritoneal macrophages isolated from WT mice and Lysm HIF1*α* lsl mice for 24 h. (d) The relative mRNA levels of M1 and M2 markers in the BMDMs isolated from the WT mice and Lysm HIF1*α* lsl mice with the treatment of LPS for 48 h. For each gene, mRNA level was normalized to the level of 18S rRNA expression. Statistical comparisons were made using two-tailed Student's *t*-test (a, b, c, and d). ^∗^*P* < 0.05 and ^∗∗^*P* < 0.01, compared with WT mice. All values were presented as mean ± SEM for *n* = 3–5 independent experiments in each group.

In this study, we used Lysm HIF1*α* lsl mice and WT mice to testify whether HIF1*α* in macrophages affects macrophage polarization as previously reported [20]. The mRNA level of *Hif1α* in bone marrow-derived macrophages (BMDMs) and peritoneal macrophages was confirmed by RT-PCR, displaying approximately threefolds of *Hif1α* expression compared with the WT mice (Figure 1(a)). Then, we examined the relative mRNA levels of M1 and M2 markers in peritoneal macrophages and BMDMs. The mRNA expressions of M1 markers, including *Il6*, *Il1b*, *Inos*, *Tnfα*, and *Cd11c*, were markedly higher in peritoneal macrophages isolated from Lysm HIF1*α* lsl mice, while the expressions of M2 markers, *Arg1*, *Cd206*, and *Chi313*, showed little difference or even lower compared with WT mice at 6 h (Figure 1(b)) and 24 h (Figure 1(c)). In BMDMs, M1 markers were highly expressed in Lysm HIF1*α* lsl mice, and the M2 markers were markedly less at the same level by comparison (Figure 1(d)). These results indicate that macrophage HIF1*α* overexpression induces M1 polarization of macrophages.

### 3.2. HIF1*α* Decreased Mitochondrial Oxidation and Promoted Glycolysis Metabolism in Macrophages

**Figure 2:**
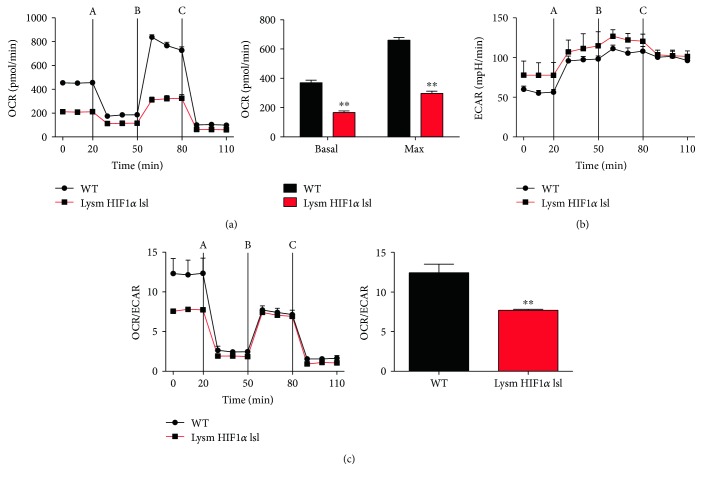
HIF1*α* decreased mitochondrial oxidation and promotes glycolysis metabolism in macrophages. (a and b) Metabolic respiratory parameters of peritoneal macrophages isolated from the WT mice and Lysm HIF1*α* lsl mice were measured with the treatment of extracellular flux analyzer: A, oligomycin; B, carbonyl cyanide p-trifluoromethoxyphenylhydrazone (FCCP); C, antimycin A and rotenone. (a) The oxygen consumption rate (OCR) value was measured at the basal level and after the treatment of A, B, and C quantitated on the right panel. Basal OCR was measured before the injection of a, and maximal OCR was calculated by subtracting the nonmitochondrial OCR from the peak OCR after the treatment of B. (b) The extracellular acidification rate (ECAR) value was calculated by the software. (c) The OCR/ECAR ratio was calculated at basal level quantitated on the right panel. Statistical comparisons were made using two-tailed Student's *t*-test (a and c). ^∗∗^*P* < 0.01 compared with WT mice. All values were presented as mean ± SEM for *n* = 9–15 independent experiments in each group.

Recent studies have indicated that the activation of macrophage polarization was marked by their metabolic programs [23, 24]. Therefore, mitochondrial oxidation was detected in peritoneal macrophages isolated from WT mice and Lysm HIF1*α* lsl mice. HIF1*α* overexpressed macrophages displayed a marked lower mitochondrial oxygen consumption rate (OCR) (Figure 2(a)) but a higher extracellular acidification rates (ECAR) (Figure 2(b)), suggesting the promotion of glycolysis metabolism. With the treatment of mitochondrial oxidative inhibitors, including carbonyl cyanide p-trifluoromethoxyphenylhydrazone (FCCP), oligomycin, antimycin A, and rotenone, the ratio of mitochondrial oxidation to glycolysis metabolism was decreased in HIF1*α* overexpression macrophages (Figure 2(c)). These data indicate that decreased mitochondrial oxidation and increased glycolysis metabolism are induced by HIF1*α* in macrophages.

### 3.3. Metabolomics Analysis Showed That HIF1*α*-Induced Glycolysis Metabolism and Pentose Phosphate Pathway and Decreased Mitochondrial Oxidation

**Figure 3:**
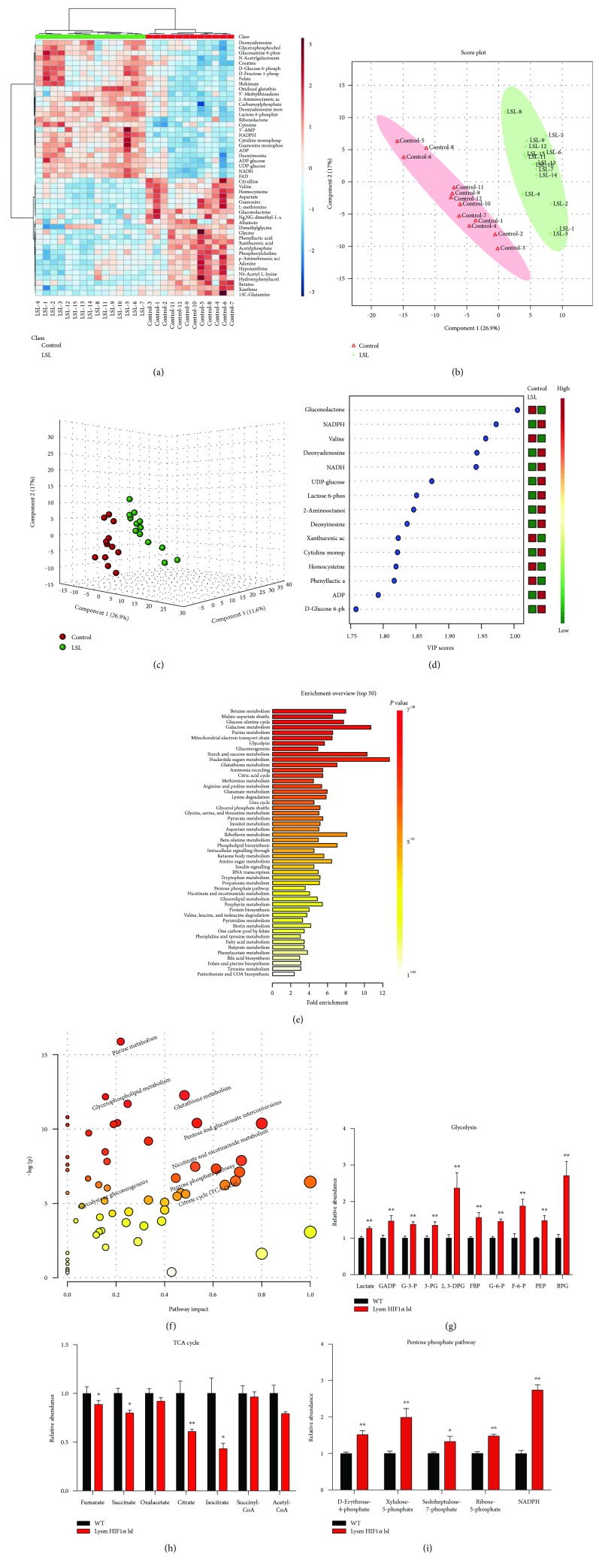
Metabolomics analysis of HIF1*α*-modified macrophage glycolysis metabolism. (a, b, c, d, e, and f) Peritoneal macrophages were isolated from the WT and Lysm HIF1*α* lsl mice. An LC-MS/MS system was used to measure the abundance of cellular metabolites. Metabolomics data were analyzed using the MetaboAnalyst website. (a) Heatmap of the intracellular metabolites generated from hierarchical clustering. Red series denoted relative high concentrations and blue series denoted relative low concentrations. (b) 2D PLS-DA score plot. (c) 3D PLS-DA score plot. (d) VIP scores. (e) Overview of metabolite enrichment in HIF1*α* overexpressed macrophages. (f) Metabolic pathway analysis of HIF1*α* overexpressed macrophages. (g, h, and i) Relative levels of metabolites in the glycolysis metabolism (g), TCA cycle (h), and pentose phosphate pathways (i). Statistical comparisons were made using two-tailed Student's *t*-test (g, h, and i). ^∗^*P* < 0.05 and ^∗∗^*P* < 0.01, compared with WT mice. All values were presented as mean ± SEM for *n* = 10–14 independent experiments in each group. FBP: fructose 1,6-bisphosphate; F-6-P: fructose-6-phosphate; GADP: glyceraldehyde-3-phosphate; G-6-P: glucose-6-phosphate; PEP: phosphoenolpyruvate; 3-PG: 3-phosphoglycerate.

The extracellular flux analysis results revealed the different metabolic mode between macrophages isolated from Lysm HIF1*α* lsl mice and WT mice. To further explore the detailed changes in metabolic profiling, metabolites were extracted from peritoneal macrophages isolated from Lysm HIF1*α* lsl mice and WT mice and analyzed using LC-MS/MS. The heatmap generated from hierarchical clustering and a partial least squares discriminant analysis (PLS-DA) plot of metabolites revealed a distinct metabolic profile in macrophages isolated from Lysm HIF1*α* lsl mice and WT mice (Figures 3(a), 3(b), and 3(c)). VIP scores extracted from the PLS-DA model demonstrated that glycolytic intermediates got relative high VIP scores (Figure 3(d)). Enrichment analysis and pathway analysis showed an apparent disparity in the glycolysis, TCA cycle, and pentose phosphate pathway (Figures 3(e) and 3(f)). Histogram analysis exhibited that the metabolite levels were increased in the glycolysis, including lactate, GADP, G-3-P, 3-PG, 2,3-DPG, FBP, G-6-P, F-6-P, PEP, and BPG (Figure 3(g)), and decreased in mitochondrial oxidation, including fumarate, succinate, citrate, and isocitrate (Figure 3(h)) in Lysm HIF1*α* lsl mice. Besides, the pentose phosphate pathway, a shunt from the glycolytic pathway, was also activated proved by the increase of d-erythrose-4-phosphate, xylulose-5-phosphate, sedoheptulose-7-phosphate, ribose-5-phosphate, and NADPH levels (Figure 3(i)). The activated pentose phosphate pathway is assumed to provide biosynthetic substrates to support macrophage growth and activation. Thus, metabolomics analysis showed an enhancement of glycolysis metabolism and pentose phosphate pathway but a decreased mitochondrial oxidation in HIF1*α* overexpressed macrophages.

### 3.4. HIF1*α*-Modified Macrophage Glycolysis Metabolism through Regulation of Glycolytic Gene Expression

**Figure 4:**
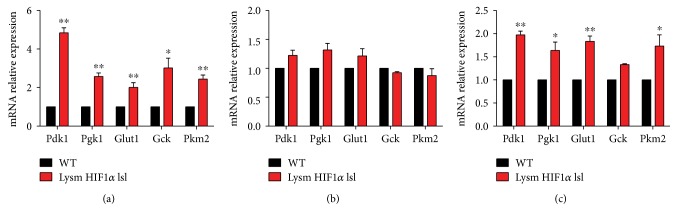
HIF1*α* activated macrophage glycolysis metabolism-related genes. (a) Relative mRNA levels of *Pdk1*, *Pgk1*, *Glut1*, *Gck*, and *Pkm2* in the peritoneal macrophages isolated from the WT mice and Lysm HIF1*α* lsl mice for 6 h. (b) Relative mRNA levels of *Pdk1*, *Pgk1*, *Glut1*, *Gck*, and *Pkm2* in the peritoneal macrophages isolated from WT mice and Lysm HIF1*α* lsl mice for 24 h. (c) Relative mRNA levels of *Pdk1*, *Pgk1*, *Glut1*, *Gck*, and *Pkm2* in the BMDMs isolated from WT mice and Lysm HIF1*α* lsl mice activated to M1 with the treatment of LPS for 48 h. For each gene, mRNA level was normalized to the level of 18S rRNA expression. Statistical comparisons were made using two-tailed Student's *t*-test. ^∗^*P* < 0.05 and ^∗∗^*P* < 0.01, compared with WT mice. All values were presented as mean ± SEM for *n* = 3–5 independent experiments in each group.

The mechanism of the glucose metabolic disparity in HIF1*α* overexpressed macrophages was explored by analyzing gene expression. mRNA expressions of some glycolytic genes, including *Pdk1*, *Pgk1*, *Glut1*, *Gck*, and *Pkm2*, were higher in peritoneal macrophages isolated from the Lysm HIF1*α* lsl mice than in the WT mice at both 6 h and 24 h (Figures 4(a) and 4(b)). The similar results were observed in BMDMs isolated from WT mice and Lysm HIF1*α* lsl mice activated to M1 with the treatment of LPS (10 ng/mL) for 48 h (Figure 4(c)).

## 4. Discussion

The liver is a site particularly enriched with innate immune cells [26] and the largest metabolic organ in the body that is responsible for various metabolic processes regulating various functions [27, 28]. Innate immune cells modify and disrupt critical processes implicated in metabolic disease. Meanwhile, metabolic stress initiates a feed-forward cycle of inflammatory responses [29]. Given that HIF1*α* is a metabolic regulator playing important roles in inflammation [30, 31], we investigated whether the regulation of cellular metabolism by HIF1*α* controls macrophage polarization and inflammation.

Our study first used HIF1*α* overexpression mice to validate the previous report that HIF1*α* promoted the accumulation of M1 macrophages [32–34]. Gene expression profiling of macrophages revealed an increase in markers of M1 macrophages and decreased or unchanged expression of M2 macrophage markers (Figure 1), supporting that HIF1 triggers macrophage polarizing to the M1 phenotype.

Recent findings suggest that cellular metabolism plays an important role during macrophage polarization [23, 35]. Classically activated macrophages secret proinflammatory mediators, accompanied with a shift from mitochondrial oxidation toward glycolysis metabolism [36]. On the contrast, alternatively activated macrophages secrete anti-inflammatory cytokines and declare an increased demand of fatty-acid oxidation [37]. Consistent with these findings, we showed that HIF1*α* overexpressed macrophages reduced cellular OCR and increased ECAR (Figure 2). The OCR/ECAR ratio was also dramatically decreased, reflecting a preference of glycolysis metabolism compared with mitochondrial oxidation in HIF1*α* overexpressed macrophages.

Macrophages are capable of coordinating their metabolic programs to adjust their immunological and bioenergetic functional properties. In our study, metabolomics profiling analysis witnessed a splendid disparation of metabolites from peritoneal macrophages isolated from WT mice and Lysm HIF1*α* lsl mice (Figure 3). Relative concentration of metabolites further demonstrated that HIF1 induced activation of glycolysis metabolism and pentose phosphate pathway and inhibited mitochondrial oxidation in macrophages in Lysm HIF1*α* lsl mice (Figure 3). Pentose phosphate pathway utilizes glucose to generate NADPH for nucleotide biosynthesis, supporting the production of reduced glutathione and therefore limits oxidative stress in M1 macrophages [38, 39]. Increased levels of pentose phosphate pathway metabolic intermediates satisfy the substrates need in HIF1*α*-prompted macrophage growth and proliferation. These data are consistent with previous studies [23, 24, 37] and lend further support to the notion that glycolysis metabolism is essential to the activation of inflammatory macrophages.

LPS-treated BMDMs were reported to tend to engage an HIF1*α*-dependent transcriptional program that is responsible for heightened glycolysis [40]. Metabolic mechanisms in HIF1*α*-deficient mice were reported to be accompanied with abolished glycolysis, decreased hepatic glucose output, and elevated gluconeogenesis [41]. On the contrast, in our study, HIF1*α* overexpression in the macrophages was accompanied with high mRNA levels of *Pdk1*, *Pgk1*, *Glut1*, *Gck*, and *Pkm2* (Figure 4), which was responsible for activated glycolysis. Heightened glycolysis may guarantee a competitive bioenergetic state and intensive energy for M1 macrophage polarization and also provide precursors for the production and secretion of proinflammatory cytokines [39, 42]. This process indicates the role of HIF1*α* in potential coordination between metabolic regulation and macrophage physiology.

## 5. Conclusions

In summary, we demonstrated that HIF1*α* activation elevates glycolysis metabolism and further induces M1 polarization of macrophages.
